# Complete Chloroplast Genome Sequence of *Hibiscus cannabinus* and Comparative Analysis of the Malvaceae Family

**DOI:** 10.3389/fgene.2020.00227

**Published:** 2020-03-17

**Authors:** Yan Cheng, Liemei Zhang, Jianmin Qi, Liwu Zhang

**Affiliations:** ^1^Key Laboratory of Ministry of Education for Genetics, Breeding and Multiple Utilization of Crops/Fujian Key Laboratory for Crop Breeding by Design, Fujian Agriculture and Forestry University, Fuzhou, China; ^2^Fujian Provincial Key Laboratory of Haixia Applied Plant Systems Biology, State Key Laboratory of Ecological Pest Control for Fujian and Taiwan Crops, College of Plant Protection, Center for Genomics and Biotechnology, Fujian Agriculture and Forestry University, Fuzhou, China

**Keywords:** *Hibiscus cannabinus*, Malvaceae, gene orientation, SSC, LSC, cp

## Abstract

Kenaf (*Hibiscus cannabinus*) is one of the most fast-growing bast in the world and belongs to the family Malvaceae. However, the systematic classification and chloroplast (cp) genome of kenaf has not been reported to date. In this study, we sequenced the cp genome of kenaf and conducted phylogenetic and comparative analyses in the family of Malvaceae. The sizes of *H. cannabinus* cp genomes were 162,903 bp in length, containing 113 unique genes (79 protein-coding genes, four rRNA genes, and 30 tRNA genes). Phylogenetic analysis indicated that the cp genome sequence of *H. cannabinus* has closer relationships with *Talipariti hamabo* and *Abelmoschus esculentus* than with *Hibiscus syriacus*, which disagrees with the taxonomical relationship. Further analysis obtained a new version of the cp genome annotation of *H. syriacus* and found that the orientation variation of small single copy (SSC) region exists widely in the family of Malvaceae. The highly variable *ycf1* and the highly conserved gene *rrn32* were identified among the family of Malvaceae. In particular, the explanation for two different SSC orientations in the cp genomes associated with phylogenetic analysis is discussed. These results provide insights into the systematic classification of the *Hibiscus* genus in the Malvaceae family.

## Introduction

Chloroplast (cp), a type of plastid characterized by its double-layer membrane and thylakoid structures, contains a high concentration of chlorophyll and is the site for the cell to conduct photosynthesis (Mustardy et al., 2008; Schattat et al., 2011), through which light energy in photons is received and converted into chemical energy via redox reactions (Jagendorf and Uribe, 1966; Neuhaus and Emes, 2003) to sustain life on earth by providing food, clothing, fuel, and oxygen (Jin and Daniell, 2015). The cp is one of two organelles that contain their own genomes (the other one is mitochondria). Since the first complete cp genome was reported in tobacco in 1986, an increasing number of cp genomes have been reported and are currently deposited in the National Center for Biotechnology Information (NCBI) database. Typically, cp genomes in angiosperms are highly conserved and have circular double-helix structures ranging from 115 to 165 kb in length, consisting of a large-single-copy region (LSC; 80–90 kb) and a small-single-copy region (SSC; 16–27 kb) separated by a pair of inverted repeats (IRs) (Ozeki et al., 1989; Palmer, 1991; Daniell et al., 2016). Generally, cp genomes of land plants contain 110–113 distinct genes, the majority of which encode proteins involved in photosynthesis (approximately 79), and the remainder of which encode transfer RNAs (approximately 30) and ribosome RNAs (4) (Jansen et al., 2005; Dong et al., 2013b; Daniell et al., 2016). Among the cp genomes of angiosperm lineages, mutations, duplications, losses, and rearrangements of genes could be observed (Lee et al., 2007). However, cp genomes are the most conserved in DNA sequences, organization, and structure compared with nuclear and mitochondrial genomes. For these reasons, the cp genomes were widely used for the phylogenetic analysis among the plant species. For example, Wu et al. (2017) elucidate the evolutionary relationships in *Commelinids* based on the sequences of 58 shared cp protein-coding genes (Wu et al., 2017), and Shen et al. (2017) revealed a sister relationship between *A. annua* and *A. fukudo* based on sequence divergence analysis of five Asteraceae species (Shen et al., 2017).

Kenaf (*Hibiscus cannabinus*) is an important natural fiber crop grown worldwide (Qi et al., 2007). Kenaf is mainly cultivated in China, India, Bangladesh, Malaysia, South Africa, Thailand, the United States, and southeastern Europe (Xiong, 2008). The fiber from kenaf is widely used for rope, coarse cloth, and paper. Recently, materials from kenaf were developed for multiple uses, such as clothing-grade cloth, insulation, engineered wood, packing material, and animal feed (Sellers, 1999). Moreover, kenaf has the potential to be developed into biofuel because of its high photosynthesis efficiency and high biomass yield. Biologically, kenaf has strong adaptability and stress resistance to drought and salinity (Cheng et al., 2004; Satya, 2012; Xu et al., 2013; Zhang et al., 2013). Because of these characteristics, kenaf is considered to be an essential crop in many countries and has received considerable attention from researchers. The family of Malvaceae, to which the genera *Hibiscus* belongs, consists of more than 100 genera, which are widely distributed in the world (Duarte et al., 2011). To date, a total of 35 cp genomes have been published in this family, among which 32 belong to the *Gossypium* genus. *Hibiscus syriacus*, *Abelmoschus esculentus*, and *Talipariti hamabo* are the species whose cp genomes have been reported.

In this study, the complete cp genome sequence of another *Hibiscus* species, *H. cannabinus*, was presented. First, the cp genome sequence was obtained, and its gene content and organization were evaluated. Second, a comparative analysis of Malvaceae species was conducted. Finally, highly variable and conserved genes of the Malvaceae cp genome were identified. The complete cp genome sequences will help to elucidate evolutionary and phylogenetic relationships in the family of Malvaceae.

## Materials and Methods

### DNA Extraction

An elite kenaf (*H. cannabinus*) cultivar, Fuhong 952, was used for cp genome sequencing. Fresh leaves from 30-day-old seedlings grown on the farm of Fujian Agriculture and Forestry University, Fuzhou, China, were collected and fine ground in liquid nitrogen. The nuclei were isolated from the sample powder with buffer A [0.1 mM Tris-HCl (pH = 8.0), 0.5 mM EDTA, 0.25 mM NaCl, and 2% PVP], and then total DNA were extracted from the nuclei using buffer B [100 mM Tris-HCl (pH = 8.0), 25 mM EDTA, 1.4 mM NaCl, 3% CTAB, 1% bisulfate, 1% ascorbic acid, 2% PVP, and 0.1% 2-ME]. The DNA procedures followed the mCTAB (modified-CTAB) method reported by Li et al. (2013).

### Chloroplast Genome Assembly and Annotation

The total cp DNA was used for Illumina library preparation, which was sequenced through Hiseq4000 platform subsequently (Dong et al., 2013a). The contigs were assembled with PE150 reads using SPAdes 3.6.1 (Bankevich et al., 2012) and SOAP denovo2 (Luo et al., 2015), respectively, and the contigs derived from the cp genome were identified by the local Blast program using Arabidopsis and Rice cp genomes as the reference (Altschul et al., 1997). The cp genome was spliced by Sequencher 4.10 using Arabidopsis cp genome as reference^1^ based on the cp contigs obtained by two assembling methods. To confirm the accuracy of the genome, the Illumina reads used for assembly were mapped back to the cp genome using Genious 8.1 (Kearse et al., 2012). To bridge the gaps, specific PCR primers were designed according to end sequences, and PCR products were sequenced with ABI 3730.

The genome was annotated using DOGMA (Dual Organellar GenoMe Annotator) software (Jansen et al., 2005). The loci and intron–exon junctions of the genes coding proteins, ribosomal RNAs (rRNAs), and transfer RNAs (tRNAs) were identified by BLASTX and BLASTN programs. The cp genome map was drawn by Organellar Genome DRAW^2^ (Lohse et al., 2013).

### Phylogenetic Analysis

The complete cp genome sequence of *H. cannabinus* was generated from this study, and the complete cp genome sequences of other species were downloaded from the NCBI website^3^. These cp sequences (Accession number: Supplementary Table S1) were used to construct phylogenetic relationships. The alignment was conducted by MAFFT (Katoh and Standley, 2013), and a phylogenetic tree was generated by MEGA 6.0 program (Tamura et al., 2013).

### Identification of SNPs and Hypervariable Regions

To identify the SNPs and hypervariable regions within the cp genome of *H. cannabinus* compared to *Gossypium raimondii* and *H. syriacus*, the cp genomes of *G. raimondii* and *H. syriacus* were aligned to the cp of *H. cannabinus* using MAFFT (Katoh and Standley, 2013). The alignment was manually adjusted using Se-Al 2.0^4^, and the nucleotide diversity (Pi) along the cp genome was calculated using DnaSP version 5 software (Librado and Rozas, 2009) with sliding window analysis. The window length was set to 800 base pairs, and the step size was set as 50 base pairs.

### Comparative Analysis of Chloroplast Genomes

The cp genomes of *H. syriacus*, *Gossypium hirsutum*, *G. raimondii* and *H. cannabinus* were used for comparative analysis. The sequences were aligned using the mVISTA program with Shuffle-LAGAN mode (Frazer et al., 2004). The cp genome of *H. syriacus* was used as a reference (Kwon et al., 2016).

## Results

### Chloroplast Genome Assembly and Annotation of *H. cannabinus*

**FIGURE 1:**
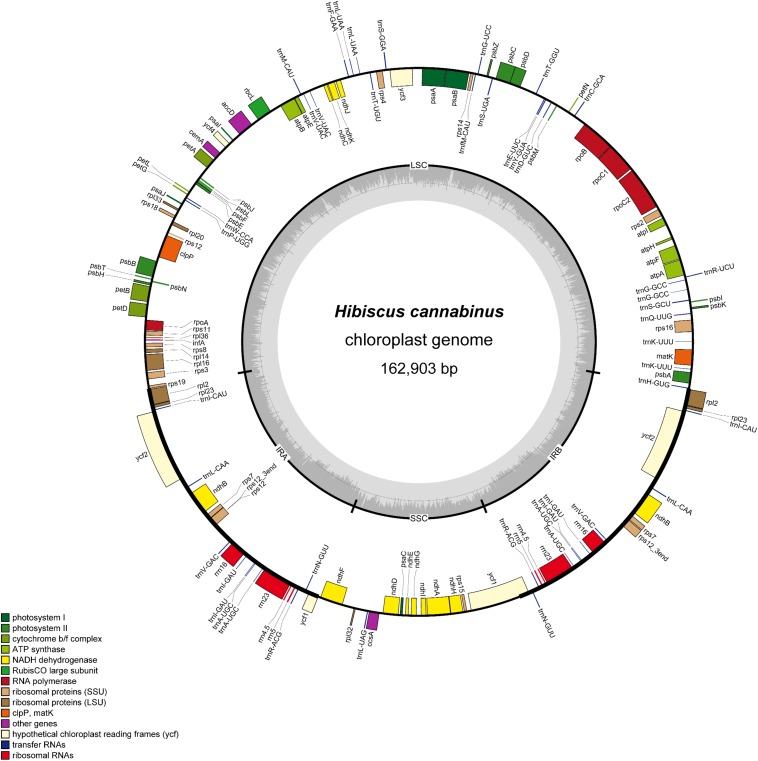
Gene map of the *H. cannabinus* chloroplast genome. The genes inside and outside of the outer circle are transcribed in the clockwise and counterclockwise directions, respectively. Genes belonging to different functional groups are shown in different colors. The inner circle represents different regions of the cp genome. IRA, inverted repeat region A; IRB, invert repeat region B; LSC, large single-copy region; SSC, small single-copy region. The line-chart in gray shows GC content along the genome.

Illumina paired-end (150 bp) sequencing produced 17442788 reads for *H. cannabinus* cp DNA, among which 907314 reads (5.20%) were derived from the cp genome with 835× coverage (Supplementary Table S2). The assembled cp genome of *H. cannabinus* (deposited in GenBank: MK404537) had a typical circular structure and conserved constitute regions (Figure 1). The complete cp genome of *H. cannabinus* is 162903 bp in size, including a pair of IRs (26533 bp each) that separate the rest of genome sequences into two single-copy regions: a LSC region (90351 bp in length) and a SSC region (19486 bp in length). The overall G + C content of the genome is 36.65%.

A total of 113 coding genes were annotated in the cp genome of *H. cannabinus*, including 30 tRNA genes, 4 rRNA genes (16S, 23S, 5S, and 4.5S), and 79 protein-coding genes (Supplementary Table S3). According to the functions of these genes, 107 were classified into three groups: (1) photosynthesis-related genes. There are 47 genes in this category, including genes encoding the large subunit of Rubisco involved in the photosynthetic electron transport chain and putative NADPH dehydrogenase genes. (2) Transcription and translation-related genes. There are 60 genes in this category, most of which are tRNA genes, and the rest are rRNA genes and genes encoding subunits of RNA polymerase and ribosome proteins. (3) Other genes. The remaining six genes with different functions are classified in this group, including four genes with known function in RAN processing (*matK*), carbon metabolism (*cemA*), fatty acid synthesis (*accD*) and proteolysis (*clpP*), and two conserved reading frames (*ycf1* and *ycf2*) encoding proteins of unknown function.

Eighteen genes were annotated with two copies located in IR regions in the cp genome of *H. cannabinus.* Most of them are related to protein translation progress, including seven transfer RNA genes, four ribosomal protein genes, and four ribosomal RNA gens. The other three are *ndhB*, encoding NADPH dehydrogenase, and two conserved reading frames with unknown function. There are 17 cp genes harbored introns, among which 15 genes contained single introns, and two genes (*ycf3*, *clpP*) contained two introns. The introns of those genes are relatively much longer the exons with the average length of 907and 204 bp, respectively. The intron of trnK-UUU is 2599 bp, which is the longest intron, and the intron of *rps12* is the shortest with the length of 536 bp. *rps12* is a trans-splicing gene in which the 5′-exon is located in LSC, and the 3′-exon is located in IR. The details of the genes structure description of these intron-contained genes were list in Supplementary Table S4.

### Simple Sequences Repeats (SSR) Analysis in *H. cannabinus*

**FIGURE 2:**
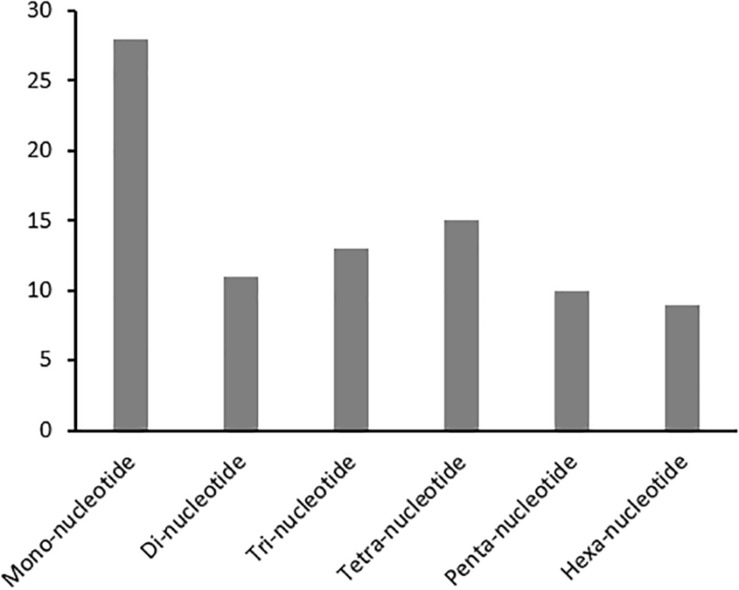
Simple Sequences Repeats (SSR) Analysis in *H. cannabinus*. The distributions of mono-, di-, tri-, tetra-, penta-, and hexa-nucleotide repeats were shown.

Developing repeatable and stable SSR markers will facilitate future genetic studies in kenaf. A set of 86 SSRs (HcSR001 through 086) specific to the *H. cannabinus* cp genome sequence with repeat lengths of at least 10 nucleotides were identified (Figure 2 and Supplementary Table S5). For example, SSR markers in *H. cannabinus* included repeat sequences with 28 mono-, 11 di-, 13 tri-, 15 tetra-, 10 penta-, and 9 hexa-nucleotide repeats. Among these, the mononucleotide repeats were highly abundant, with frequencies of 32.56% in *H. cannabinus*.

### Phylogenetic Analysis of Chloroplast Genomes of Malvaceae Plants

**FIGURE 3:**
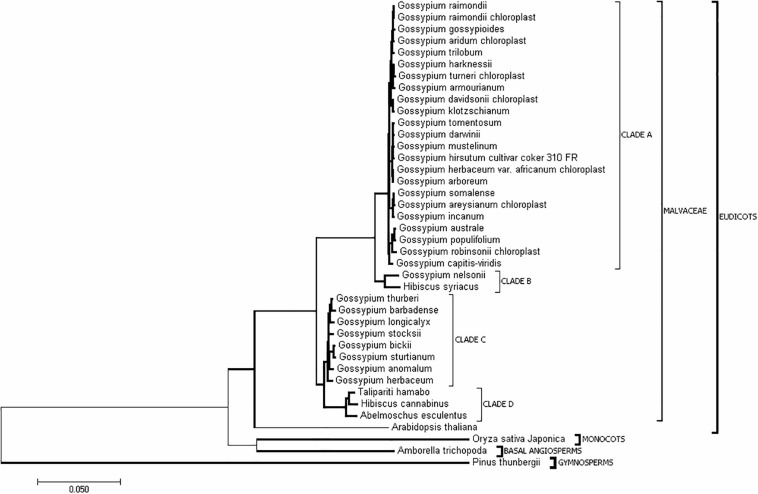
Evolutionary relationships of taxa. The evolutionary history was inferred using the Neighbor-Joining method (Saitou and Nei, 1987). The optimal tree with the sum of branch length = 0.92533808 is shown. The tree is drawn to scale, with branch lengths in the same units as those of the evolutionary distances used to infer the phylogenetic tree. The evolutionary distances were computed using the maximum composite likelihood method (Tamura et al., 2004) and are in units of the number of base substitutions per site. The analysis involved 40 nucleotide sequences. All ambiguous positions were removed for each sequence pair. There were 226151 positions in total in the final dataset. Evolutionary analyses were conducted in MEGA7 (Kumar et al., 2016).

In addition to *H. cannabinus*, there are 35 species with complete cp genome sequences in the public database (Supplementary Table S1). To understand the evolutionary relationships among Malvaceae species, the complete cp genome sequences of these Malvaceae species and outgroup species (*Pinus thunbergii*, *Oryza sativa*, and *Zea mays*) were downloaded from NCBI. The phylogenetic tree was constructed using Mega 6.0 (Figure 3 and Supplementary Figure S1). As shown in Figure 3, the cp genome sequence of *H. cannabinus* has closer relationships with *T. hamabo* and *A. esculentus* than with *H. syriacus*. Among the *Gossypium* species, *G. barbadense*, *G. thurberi*, *G. anomalum*, *G. herbaceum*, *G. longicalyx*, and *G. stocksii* have the highest similarity with *H. cannabinus*, while *G. raimondii* and *G. hirsutum* (*G. hirsutum cultivar_coker_310_FR*) has the lowest similarity with *H. cannabinus.*

### Comparative Analysis of Malvaceae Chloroplast Genomes

**FIGURE 4:**
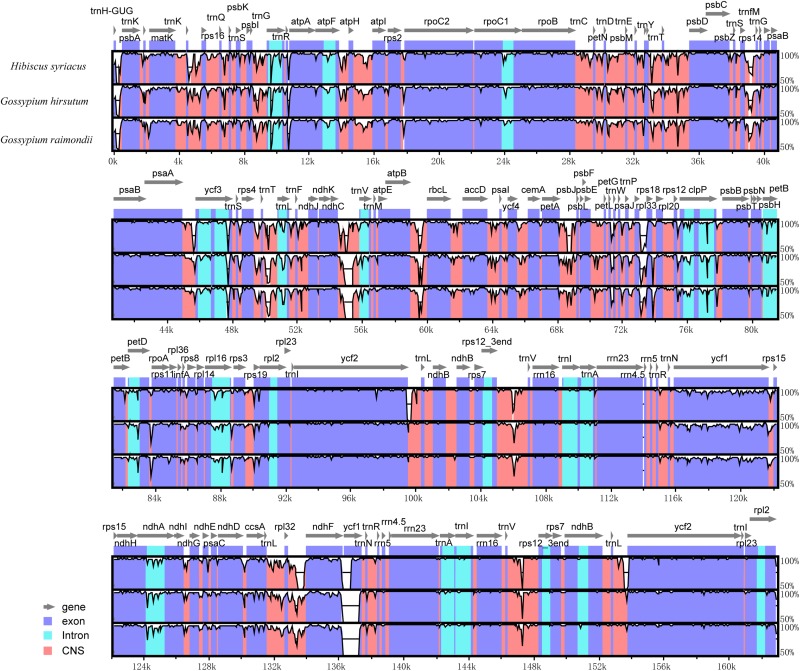
Sequence identity plot comparing the chloroplast genome of *Hibiscus cannabinus. Hibiscus syriacus*, *Gossypium hirsutum*, and *Gossypium raimondii*. The vertical scale indicates the percentage of identity, ranging from 50 to 100%. The horizontal axis indicates the coordinates within the cp genome. Genome regions are color-coded as protein-coding, rRNA, tRNA, intron, and conserved non-coding sequences (CNS).

*Further*, a comparative analysis of cp genomes between *H. cannabinus* species and other widely cultivated species of Malvaceae family notably, *H. syriacus*, *G. hirsutum*, and *G. raimondii* was conducted, and the identity among the entire cp genomes was analyzed through sequence identity plots (Figure 4). The IRs of three cp genomes showed relatively lower identity than the LSC and SSC regions. By considering different functional regions, the conserved non-coding sequences (CNS) regions showed the highest variation. However, the exon regions of four cp genomes exhibited relatively higher conservation than the CNS and intron regions. In the IRs, the *rrn23* gene with two copies showed the highest identity within four cp genomes. Among the hypervariable regions, the *rpl32-trnN* sequences showed the highest variation, and the *ycf1* gene in this region is the most divergent. In comparison with *H. cannabinus*, the cp genomes of *H. syriacus*, *G. hirsutum*, and *G. raimondii* have deletions in the large copy of the *ycf1* locus.

To confirm these findings, a similar comparative analysis with 10 expanded taxa representing different clades in the phylogenetic tree was performed (Supplementary Figure S2). As shown in Supplementary Figure S2, *rrn32* was the most conserved gene, while *ycf1* was the highly variable gene. Moreover, seven variation sites along with *rrn23* genes were observed (Supplementary Table S6). Five *Gossypium* species showed a fully sequence identity with *H. cannabinus*, while two *Gossypium* cultivars harbored one nucleotide variation. *A. esculentus* (with five SNPs) was the most variable species compared with *H. cannabinus*. Because of the highly variable characteristics of *ycf1* in the cp genomes of the Malvaceae family, a phylogenetic tree was generated based on the sequence of *ycf1*. As shown in Supplementary Figure S3, most *Gossypium* species fall into Clade A, and the remaining species in the family of Malvaceae fall into Clade B. Interestingly, *G. nelsonii* also belongs to Clade B, although it has a closer relationship with most of the *Gossypium* species based on the whole cp genome sequences (Figure 3).

### Chloroplast Genome Comparative Analysis Between *H. cannabinus* and *G. raimondii*

**FIGURE 5:**
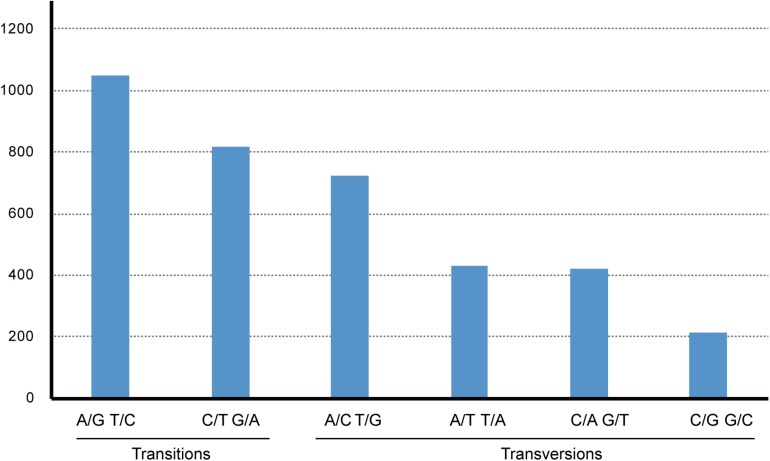
The pattern of nucleotide substitutions between *Hibiscus cannabinus* and *Gossypium raimondii* chloroplast genomes. The patterns were divided into six types as indicated by the six non-stand-specific base-substitution types (i.e., numbers of considered G to A and C to T sites for each respective set of associated variation types). The cp of *Gossypium raimondii* was used as a reference.

**FIGURE 6:**
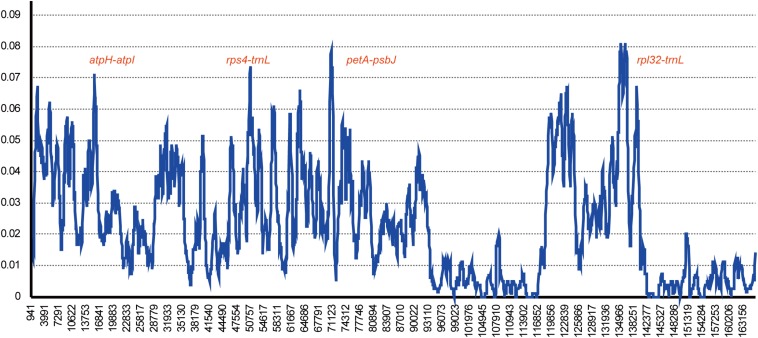
Chloroplast genome comparative analysis between *H. cannabinus* and *G. raimondii.* Sliding window plots of nucleotide diversity (π) across the complete cp genomes of *Hibiscus cannabinus* and *Gossypium raimondii* (window length: 600 bp, step size: 100 bp). *Y*-axes: nucleotide diversity (π) of each window; *X*-axes: the position of the midpoint of a window.

*Gossypium raimondii* is an important cotton crop in agriculture with a diploid genome. Its genome was first deciphered in 2012 (Wang et al., 2012) among the Malvaceae plants. The previous phylogenetic analysis in this study showed that the genome sequence of *G. raimondii* has relatively lower similarity with *H. cannabinus*. For those reasons, a comparative analysis between the cp genome sequences of *G. raimondii* and *H. cannabinus* was conducted. As shown in Figure 5, 3655 substitutions, including transitions (*Ts*) and transversions (*Tv*), were detected. The variability in the whole genome was also quantified using the average nucleotide diversity (π). Generally, the IRa and IRb exhibited lower variability than the LSC and SSC regions. Four regions (*atpH–atpI*, *rps4–trnL*, *petA–psbJ*, and *rpl32-trnN*) showed relatively high variation between two cp genomes (π > 0.7, Figure 6).

### Chloroplast Genome Comparative Analysis Between *H. cannabinus* and *H. syriacus*

**FIGURE 7:**
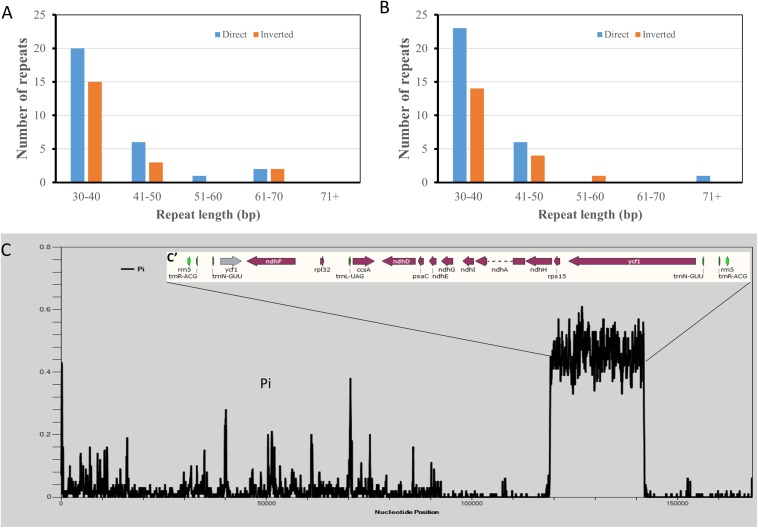
Comparative analysis of chloroplast genomic DNA sequences of *Hibiscus cannabinus* and *Hibiscus syriacus.* **(A)** The number of repeated sequences in *Hibiscus cannabinus*. **(B)** The number of repeated sequences *Hibiscus syriacus*. **(C)** Sliding window plots of nucleotide diversity (π) across the complete cp genomes of *Hibiscus cannabinus* and *Gossypium raimondii* (window length: 600 bp, step size: 100 bp). *Y*-axes: nucleotide diversity (π) of each window; *X*-axes: the position of the midpoint of a window.

Two *Hibiscus* species, *H. syriacus* and *H. cannabinus* have the closest taxonomical relationship. However, phylogenetic analysis based on cp genome sequences showed the opposite tendency. To reveal the reason for this contradiction, tentative comparative analyses were performed between the two cp genomes. Repeat analysis identified 49 repeats in each cp genome. As a result, 30 direct repeats and 19 inverted repeats were detected in the cp genome of *H. cannabinus*, while 29 direct repeats and 20 inverted repeats were detected in that of *H. syriacus.* The *H. cannabinus* cp genome contains five repeats exceeding 50 bp (Figure 7A and Supplementary Table S7), while *H. cannabinus* has only two repeats exceeding 50 bp (Figure 7B and Supplementary Table S7). However, a significant difference in the cp genomes between *H. syriacus* and *H. cannabinus* was not observed in this analysis. We then aligned the DNA sequences of the two cp genomes and subsequently detected the variation regions using DNAsp 6.0. Interestingly, unexpectedly highly varied regions (*H. cannabinus*, 118942-141948; 115805-141948, *H. syriacus*) were detected between the SSCs of the two species (Figure 7C).

### Opposite SSC Orientations in *H. syriacus* and *H. cannabinus*

To uncover the high variation of SSC between *H. syriacus* and *H. cannabinus*, the cp genome sequence (KP688069) of *H. syriacus* was downloaded from NCBI, and the gene maps corresponding to SSC regions were drawn using Organelle Draw software. As shown in Supplementary Figure S4, the gene layouts within SSC regions of the two cp genomes are in the opposite orientations, indicating that there is an inverted arrangement of SSC between the two cp genomes. Moreover, we only found a small number of annotated genes within the IRs of *H. syriacus* cp, indicating that the original annotation of this cp genome might not be accomplished completely. To this end, we reannotated the NCBI sequence KP688069 by DOGMA software, resulting in a new version of the cp genome annotation of *H. syriacus* (KP688069.2). As shown in Supplementary Figure S5, the original annotation of KP688069 was incomplete, especially in the inverted regions. We annotated most of the missing genes in IRs, while the longer copy of *ycf1* still lacked in the SSC region. The cp map of the newly annotated version clearly showed the inverted orientation of SSC of *H. syriacus* in comparison with that of *H. cannabinus*.

## Discussion

### Malvaceae Chloroplast Genomes Are Conserved in Genome Structure and Gene Content

Malvaceae is a family of flowering plants and is estimated to contain 244 genera with 4225 known species over the world (Christenhusz and Byng, 2016). Among these species, okra, cotton, cacao, and durian are well-known economic crops. A total of 35 cp genomes in the Malvaceae family have been reported to date, 32 of which belong to *Gossypium*. *Gossypium* species have received considerable attention because they are the essential fiber resources for human beings. In this study, we assembled the cp genome of *H. cannabinus*, which is the second cp genome reported in *Hibiscus*. The length of the cp genome is 162903 bp (Figure 1), consistent with most of the cp genomes in angiosperms (115–165 kb), and the number of coding genes is 113 (Supplementary Table S3), similar to the number of previously reported cp genomes. The comparative analysis also showed the conserved characteristics of the Malvaceae family.

As is well-known, the cp and mitochondrial genomes possess matrilineal inheritance in plants. Unlike the nuclear genomes, the cp and mitochondrial DNA of offspring are from only the maternal parent, and deficient recombination occurred during meiosis. Therefore, cp and mitochondrial genomes are more conserved than nuclear genomes. It has been reported that the nucleotide substitution rate of cp genes is lower than that of the nuclear gene, but higher than that of mitochondrial genes (Wolfe et al., 1987). On the other hand, the cp genome of higher plants has a highly conserved organization with a single circular DNA composed of two copies of an inverted repeat that separates the LSC region and SSC region, and the gene component and organization of cp were considerably more conserved than that of mitochondria. Gene annotation and functional classification also showed that *H. cannabinus* cp has a typical LSC–IR–SSC–IR organization (Figure 1) and the same gene categories as other plants (Supplementary Table S3).

### Opposite Orientations of SSC Might Be Synchronously Present in Chloroplasts

*Hibiscus syriacus* was the first *Hibiscus* species with a reference cp genome. Unexpectedly, the cp genome of *H. syriacus* was not completely annotated according to the data deposited in NCBI (KP688069). Several genes were missing in the IRs (Supplementary Figures S4, S5). Reannotation of *H. syriacus* and annotation of *H. cannabinus* will provide insights into the cp genomes of the *Hibiscus* genus in the Malvaceae family. Extensively comparative analysis observed the inverted orientations of the SSC region between the two *Hibiscus* cp genomes. *H. cannabinus* has the same orientation as *Arabidopsis* (+), while *H. syriacus* has the inverted orientation of SSC (−). Further analysis showed that the inverted orientation of SSC exists widely in the Malvaceae family. Among the 36 species in the Malvaceae family, 25 species in clades A and B have an inverted (−) SSC orientation, and the remaining 16 species in clades C and D have the same SSC orientation (+) as *Arabidopsis* (Figure 3). Interestingly, the different orientation of SSC in the cp genomes seemed to be associated with different groups in the phylogenetic analysis. The causes of this phenomenon will receive considerable attention by plant researchers. There might be two reasons for such an outcome of the relationship between the orientation of SSC in the cp genomes and groups in the phylogenetic analysis. One reason is that there are two different orientations of SSC in one plant, which resulted from the recombination events between the two IRs. The other possibility is that without knowing the actual orientation of SSC in one plant based on the sequences from the first- and second-generation sequencing technologies, the length of IRs is >20 kb and exceeds the maximal read lengths of first- and second-generation sequencing. In our opinion, to assemble the cp genome with actual SSC orientation, one intact sequence read spanning at least one IR is required to fix the relative directions of LSC and SSC.

### Chloroplastic *ycf1* Could Be Used as a Molecular Marker for Malvaceae Plants

Comparative analysis showed that different types of DNA fragments have different extents of sequence variation (Supplementary Figure S2). Generally, the variation of introns was higher than exons but lower than CNS regions; this tendency was also found in other analyses (Dong et al., 2013b; Nazareno et al., 2015; Yao et al., 2015). The cp genome of *H. cannabinus* and comparative analysis with cp genomes of other Malvaceae species provides insights into phylogenetic and evolutionary studies in this family. To address the question of whether there are any highly variable and conserved regions in the cp genome of Malvaceae, the 10 cp genome sequences located in different clades in the evolutionary phylogenetic tree were compared. Among the annotated genes, *ycf1* had the highest variation, while *rrn32* was the most conserved gene in the Malvaceae family. Huang et al. (2014) identified 15 molecular markers with >1.5% sequence divergence based on five *Camellia* cp genomes, which may be useful for further phylogenetic analysis and species identification of *Camellia*. Xu et al. (2012) compared sequence variations of 13 *Gossypium* cp genomes and found that the cp divergence was approximately 0.00159–0.00454 within allotetraploids of *Gossypium*. Middleton et al. (2014) developed InDel and SNP markers from 12 *Triticeae* cp species and estimated that barley, rye, and wheat diverged approximately 8.5 million years ago. These results indicate that molecular markers based on *ycf1* are highly useful in evolution analysis and systematic classification in the Malvaceae family.

## Data Availability Statement

The assembled chloroplast genome of *H. cannabinus* was deposited in GenBank: MK404537. The sequences is available on NCBI now https://www.ncbi.nlm.nih.gov/nuccore/MK404537.1/.

## Author Contributions

YC and LieZ performed most of the research and drafted the manuscript, and performed the data analysis. LiwZ carried out the grafting experiments. JQ revised the manuscript. LiwZ designed the experiments, supervised the study, and revised the manuscript.

## Conflict of Interest

The authors declare that the research was conducted in the absence of any commercial or financial relationships that could be construed as a potential conflict of interest.

## Footnotes

1 http://www.genecodes.com

2 http://ogdraw.mpimp-golm.mpg.de/index.shtml

3 https://www.ncbi.nlm.nih.gov/

4 http://tree.bio.ed.ac.uk/software/seal/
