# Supplementary material for: Complete Chloroplast Genome Sequence of Hibiscus cannabinus and Comparative Analysis of the Malvaceae Family
Source: Front Genet. 2020 Mar 17;11:227. doi: 10.3389/fgene.2020.00227 (PMC7090147; doi:10.3389/fgene.2020.00227)
Supplement: Supplementary file 1 [file Data_Sheet_1.DOCX]

**Table S1.** Information of reported chloroplast genomes in Malvaceae.

| Organism Name | BioProject | Size(Mb) | GC% | Type | Replicons | CDS |
| --- | --- | --- | --- | --- | --- | --- |
| *Abelmoschus esculentus* | PRJNA394355 | 0.163121 | 36.7365 | chloroplast | NC_035234.1/KY635876.1 | 87 |
| *Gossypium anomalum* | PRJNA232874 | 0.159507 | 37.3256 | chloroplast | NC_023213.1/JF317351.1 | 86 |
| *Gossypium arboreum* | PRJNA335838 | 0.16023 | 37.2296 | chloroplast | NC_016712.1/HQ325740.1 | 83 |
| *Gossypium areysianum* | PRJNA170282 | 0.159572 | 37.3687 | chloroplast | NC_018112.1/JN019795.1 | 83 |
| *Gossypium aridum* | PRJNA362452 | 0.160257 | 37.2645 | chloroplast | NC_033396.1/KP170502.1 | 84 |
| *Gossypium armourianum* | PRJNA362377 | 0.160068 | 37.2904 | chloroplast | NC_033400.1/KP221926.1 | 84 |
| *Gossypium australe* | PRJNA362448 | 0.159578 | 37.1561 | chloroplast | NC_033401.1/KP221928.1 | 84 |
| *Gossypium barbadense* | PRJNA18453 | 0.160317 | 37.2294 | chloroplast | NC_008641.1/AP009123.1 | 84 |
| *Gossypium bickii* | PRJNA232867 | 0.159422 | 37.1969 | chloroplast | NC_023214.1/JF317352.1 | 87 |
| *Gossypium capitis-viridis* | PRJNA170280 | 0.159467 | 37.3206 | chloroplast | NC_018111.1/JN019794.1 | 83 |
| *Gossypium darwinii* | PRJNA81249 | 0.160378 | 37.2339 | chloroplast | NC_016670.1/HQ325741.1 | 83 |
| *Gossypium davidsonii* | PRJNA362453 | 0.160072 | 37.3101 | chloroplast | NC_033395.1/KP170501.1 | 84 |
| *Gossypium gossypioides* | PRJNA163975 | 0.159959 | 37.3121 | chloroplast | NC_017894.1/HQ901195.1 | 83 |
| *Gossypium harknessii* | PRJNA362196 | 0.160129 | 37.2968 | chloroplast | NC_033333.1/KP221927.1 | 84 |
| *Gossypium herbaceum* | PRJNA232880 | 0.16014 | 37.308 | chloroplast | NC_023215.1/JF317353.1 | 86 |
| *Gossypium herbaceum subsp. africanum* | PRJNA81293 | 0.160315 | 37.218 | chloroplast | NC_016692.1/HQ325742.1 | 83 |
| *Gossypium hirsutum* | PRJNA320881 | 0.160301 | 37.2449 | chloroplast | NC_007944.1/DQ345959.1 | 83 |
| *Gossypium incanum* | PRJNA170283 | 0.159205 | 37.392 | chloroplast | NC_018109.1/JN019792.1 | 83 |
| *Gossypium klotzschianum* | PRJNA362454 | 0.160097 | 37.3036 | chloroplast | NC_033394.1/KP170500.1 | 84 |
| *Gossypium longicalyx* | PRJNA232876 | 0.160241 | 37.242 | chloroplast | NC_023216.1/JF317354.1 | 86 |
| *Gossypium mustelinum* | PRJNA81327 | 0.160313 | 37.2209 | chloroplast | NC_016711.1/HQ325743.1 | 83 |
| *Gossypium nelsonii* | PRJNA362447 | 0.162316 | 36.8011 | chloroplast | NC_033399.1/KP221925.1 | 84 |
| *Gossypium populifolium* | PRJNA362446 | 0.159444 | 37.1961 | chloroplast | NC_033398.1/KP221924.1 | 84 |
| *Gossypium raimondii* | PRJNA282644 | 0.160161 | 37.3075 | chloroplast | NC_016668.1/HQ325744.1 | 83 |
| *Gossypium raimondii* | PRJNA320118 | 0.160161 | 37.3075 | chloroplast | NC_016668.1/HQ325744.1 | 83 |
| *Gossypium robinsonii* | PRJNA170278 | 0.159849 | 37.1601 | chloroplast | NC_018113.1/JN019791.1 | 83 |
| *Gossypium somalense* | PRJNA170281 | 0.159539 | 37.3652 | chloroplast | NC_018110.1/JN019793.1 | 83 |
| *Gossypium stocksii* | PRJNA232863 | 0.159039 | 37.4128 | chloroplast | NC_023217.1/JF317355.1 | 86 |
| *Gossypium sturtianum* | PRJNA232882 | 0.159627 | 37.1334 | chloroplast | NC_023218.1/JF317356.1 | 86 |
| *Gossypium thurberi* | PRJNA63381 | 0.160264 | 37.218 | chloroplast | NC_015204.1/GU907100.1 | 85 |
| *Gossypium tomentosum* | PRJNA81289 | 0.160433 | 37.2043 | chloroplast | NC_016690.1/HQ325745.1 | 83 |
| *Gossypium trilobum* | PRJNA362378 | 0.160109 | 37.2902 | chloroplast | NC_033397.1/KP170503.1 | 84 |
| *Gossypium turneri* | PRJNA281916 | 0.159927 | 37.2614 | chloroplast | NC_026835.1/JQ742090.1 | 83 |
| *Hibiscus syriacus* | PRJNA281849 | 0.161019 | 36.8298 | chloroplast | NC_026909.1/KP688069.1 | 79 |
| *Talipariti hamabo* | PRJNA321894 | 0.161729 | 36.8988 | chloroplast | NC_030195.1/KR259988.1 | 85 |

**Note:** The information listed in the table was download from NCBI (https://www.ncbi.nlm.nih.gov/).

**Table S2.** The statistic information of chloroplast genome assembly.

| Species | *H. cannabinus* |
| --- | --- |
| Total reads | 17442788 |
| Chloroplastic reads | 907314 |
| Percentage of chloroplstic reads (%) | 5.2 |
| Chloroplastic reads coverage (×) | 835 |
| Chlorplast genome size (bp) | 162903 |
| Large single-copy size (bp) | 90351 |
| Large single-copy size (bp) | 19486 |
| Inverted repeat (bp) | 26533 |

**Table S3.** Chloroplast encoding genes of *H. cannabinus.*

| Category for genes |  | Group of gene | Name of gene |
| --- | --- | --- | --- |
| Photosynthesis related genes |  | Rubisco | *rbcL* |
|  |  | Photosystem Ⅰ | *psaA,psaB,psaC,psaI,psaJ* |
|  |  | Assembly/stability of photosystem Ⅰ | **ycf3,ycf4* |
|  |  | Photosystem Ⅱ | *psbA,psbB,psbC,psbD,psbE,psbF,psbH,psbI,psbJ,psbK,psbL,psbM,psbN,psbT,psbZ* |
|  |  | ATP synthase | *atpA, atpB, atpE, *atpF, atpH, atpI* |
|  |  | cytochrome b/f complex | *petA, *petB, *petD, petG, petL, petN* |
|  |  | cytochrome c synthesis | *ccsA* |
|  |  | NADPH dehydrogenase | **ndhA, *ndhB, ndhC, ndhD, ndhE, ndhF, ndhG, ndhH, ndhI, ndhJ, ndhK* |
| Transcription and translation related genes |  | transcription | *rpoA, rpoB, rpoC1, rpoC2* |
|  |  | ribosomal proteins | *rps2, rps3, rps4, rps7, rps8, rps11, *rps12, rps14,rps15, rps16, rps18, rps19,*rpl2, rpl14, *rpl16, rpl20, rpl22, rpl23, rpl32, rpl33,rpl36* |
|  |  | translation initiation factor | *infA* |
| RNA genes |  | ribosomal RNA | *rrn5, rrn4.5, rrn16, rrn23* |
|  |  | transfer RNA | **trnA-UGC, trnC-GCA, trnD-GUC, trnE-UUC, trnF-GAA, trnG-UCC, *trnG-GCC, trnH-GUG, trnI-CAU, *trnI-GAU,*trnK-UUU, trnL-CAA, *trnL-UAA, trnL-UAG, trnfM-CAUI,trnM-CAU, trnN-GUU, trnP-UGG, trnQ-UUG,trnR-ACG, trnR-UCU, trnS-GCU, trnS-GGA, trnS-UGA, trnT-GGU,trnT-UGU, trnV-GAC, *trnV-UAC, trnW-CCA, trnY-GUA* |
| Other genes |  | RNA processing | *matK* |
|  |  | carbon metabolism | *cemA* |
|  |  | fatty acid synthesis | *accD* |
|  |  | proteolysis | **clpP* |
| Genes of unknown function |  | conserved reading frames | *ycf1, ycf2* |

Intron-containing genes are marked by asterisks (*).

**Table S4.** The details of gens with introns.

| Gene | Region | Stand | Copy Number | Intron Number | Length (bp) | | | | |
| --- | --- | --- | --- | --- | --- | --- | --- | --- | --- |
|  |  |  |  |  | Exon 1 | Inton 1 | Exon 2 | Inton 2 | Exon 3 |
| ycf3 | LSC | complement | 1 | 2 | 126 | 870 | 228 | 793 | 153 |
| atpF | SSC | complement | 1 | 1 | 159 | 804 | 411 |  |  |
| petB | LSC | (+) strand | 1 | 1 | 6 | 825 | 642 |  |  |
| petD | LSC | (+) strand | 1 | 1 | 8 | 753 | 481 |  |  |
| ndhA | SSC | complement | 1 | 1 | 540 | 1158 | 540 |  |  |
| ndhB | IR | (+) strand | 2 | 1 | 777 | 683 | 756 |  |  |
| rps12 | lSC | complement | 1 | 0 | 114 |  |  |  |  |
| rps12_3end | IR | complement | 2 | 1 |  |  | 231 | 536 | 27 |
| rpl2 | IR | complement | 2 | 1 | 391 | 698 | 434 |  |  |
| rpl16 | LSC | complement | 1 | 1 | 9 | 1159 | 399 |  |  |
| trnA-UGC | IR | complement | 2 | 1 | 38 | 794 | 35 |  |  |
| trnG-GCC | LSC | complement | 1 | 1 | 48 | 901 | 24 |  |  |
| trnI-GAU | IR | (+) strand | 2 | 1 | 42 | 953 | 35 |  |  |
| trnK-UUU | LSC | complement | 1 | 1 | 37 | 2599 | 35 |  |  |
| trnL-UAA | LSC | (+) strand | 1 | 1 | 37 | 583 | 50 |  |  |
| trnV-UAC | LSC | complement | 1 | 1 | 38 | 605 | 37 |  |  |
| clpP | LSC | complement | 1 | 2 | 69 | 966 | 294 | 653 | 228 |

LSC: Large single copy region; IR: Inverted repeat region; SSC: Small single copy region.

**Table S5.** Location of simple sequence repeats based on in the chloroplast genome of *Hibiscus cannabinus.*

Note: Table S5 was provided as an individual Excel file.

**Table S6.** Nucleotide variations of *rrn32* among 11 representative taxa in Malvaceae

| No. | Species | Accession | 170 | 311 | 329 | 338 | 1120 | 2091 | 2801 |
| --- | --- | --- | --- | --- | --- | --- | --- | --- | --- |
| 1 | *Hibiscus cannabinus* | K06 | T | G | C | G | C | C | G |
| 2 | *Talipariti hamabo* | KR259988.1 | T | G | C | G | C | T | G |
| 3 | *Abelmoschus esculentus* | KY635876.1 | C | T | A | T | C | T | G |
| 4 | *Gossypium barbadense* | AP009123.1 | T | G | C | G | T | C | A |
| 5 | *Gossypium herbaceum* | JF317353.1 | T | G | C | G | C | C | G |
| 6 | *Hibiscus syriacus* | KP688069.1 | T | G | C | G | C | T | G |
| 7 | *Gossypium nelsonii* | KP221925.1 | T | G | C | G | C | T | G |
| 8 | *Gossypium herbaceum subsp.Africanum* | HQ325742.1 | T | G | C | G | C | C | G |
| 9 | *Gossypium arboreum* | HQ325740.1 | T | G | C | G | C | C | G |
| 10 | *Gossypium hirsutum coker 310 FR* | DQ345959.1 | T | G | C | G | C | C | G |
| 11 | *Gossypium raimondii* | HQ325744.1 | T | G | C | G | C | C | G |

The red letter showed the nucleotide variation of *rrn32* compared with *Hibiscus cannabinus*. The variation sites were shown in the first row.

**Table S7.** Sequence repeats in *Hibiscus syriacus* and *Hibiscus cannabinus*

| Species | Repeat Type |  | Repeat Length (bp) | | | | | Total |
| --- | --- | --- | --- | --- | --- | --- | --- | --- |
|  |  |  | 30-40 | 41-50 | 51-60 | 61-70 | 71+ |  |
| *Hibiscus syriacus* | Direct |  | 23 | 6 | 0 | 0 | 1 | 30 |
|  | Inverted |  | 14 | 4 | 1 | 0 | 0 | 19 |
| *Hibiscus cannabinus* | Direct |  | 20 | 6 | 1 | 2 | 0 | 29 |
|  | Inverted |  | 15 | 3 | 0 | 2 | 0 | 20 |

The statistical data of sequence repeats analysis consistent to Figure 5a, b.

**Supplementary figures**





**Figure S1.** Evolutionary relationships of Malvaceae taxa based on the chloroplast genome sequences.

The evolutionary history was inferred using the Neighbor-Joining method ([Saitou & Nei, 1987](#_ENREF_4))The bootstrap consensus tree inferred from 1000 replicates ([Felsenstein, 1985](#_ENREF_1)) is taken to represent the evolutionary history of the taxa analyzed ([Felsenstein, 1985](#_ENREF_1)). Branches corresponding to partitions reproduced in less than 50% bootstrap replicates are collapsed. The percentage of replicate trees in which the associated taxa clustered together in the bootstrap test (1000 replicates) are shown next to the branches ([Felsenstein, 1985](#_ENREF_1)). The evolutionary distances were computed using the Maximum Composite Likelihood method ([Tamura et al., 2004](#_ENREF_5)) and are in the units of the number of base substitutions per site. The analysis involved 40 nucleotide sequences. All ambiguous positions were removed for each sequence pair. There were a total of 226151 positions in the final dataset. Evolutionary analyses were conducted in MEGA7 ([Kumar et al., 2016](#_ENREF_2)).


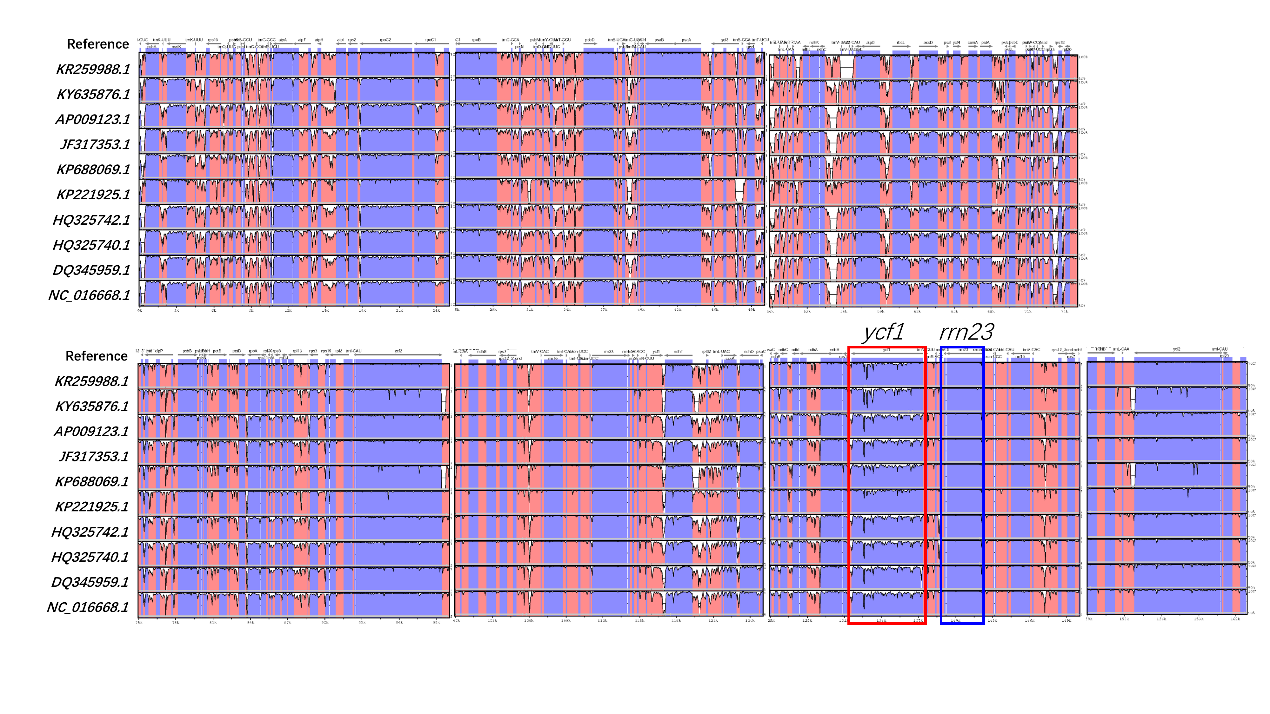


**Figure S2.** Sequence identity plot comparing the chloroplast genome of *Hibiscus cannabinus* and the other 10 representative species in Malvaceae.

The vertical scale indicates the percentage of identity, ranging from 50% to 100%. The horizontal axis indicated the coordinates within the chloroplast genome. Genome regions are color coded as protein coding, rRNA, tRNA, intron, and conserved non-coding sequences (CNS).

**

**

**Figure S3.** Evolutionary relationships of 10 Malvaceae taxa based on *ycf1* gene sequences.

The evolutionary history was inferred using the Neighbor-Joining method ([Saitou & Nei, 1987](#_ENREF_4)). The optimal tree with the sum of branch length = 0.05542797 is shown. The percentage of replicate trees in which the associated taxa clustered together in the bootstrap test (1000 replicates) are shown next to the branches ([Felsenstein, 1985](#_ENREF_1)). The tree is drawn to scale, with branch lengths in the same units as those of the evolutionary distances used to infer the phylogenetic tree. The evolutionary distances were computed using the Maximum Composite Likelihood method ([Saitou & Nei, 1987](#_ENREF_4)) and are in the units of the number of base substitutions per site. The analysis involved 11 nucleotide sequences. Codon positions included were 1st+2nd+3rd+Noncoding. All ambiguous positions were removed for each sequence pair. There were a total of 5817 positions in the final dataset. Evolutionary analyses were conducted in MEGA7 ([Kumar et al., 2016](#_ENREF_2)).


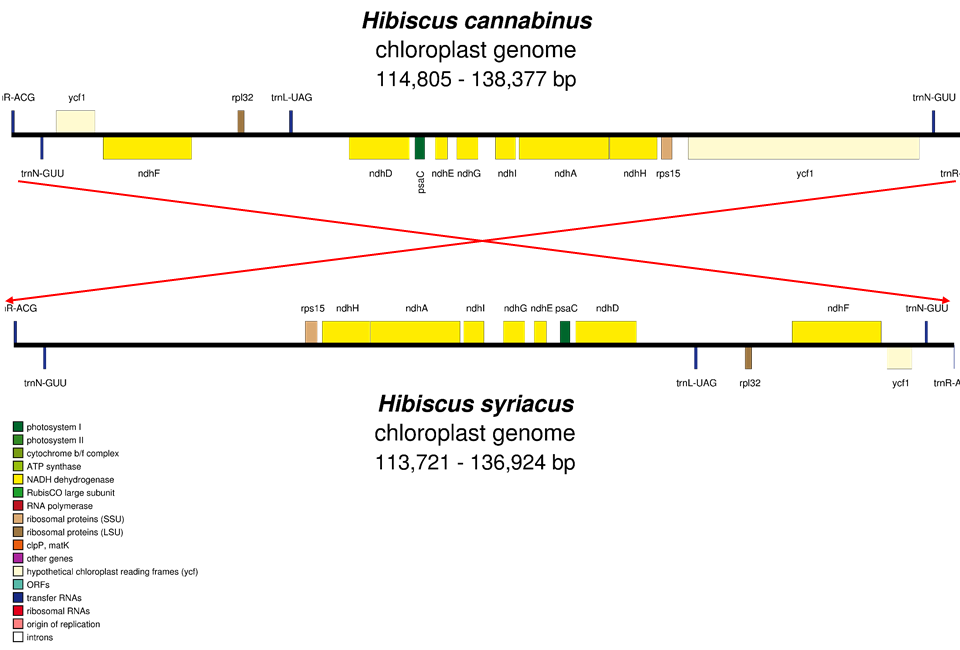


**Figure S4.** Chloroplast LSC regions of *Hibiscus cannabinus* and *Hibiscus syriacus.*

The genes transcribed in the forward and reverse directions are shown on the top and blow, respectively.


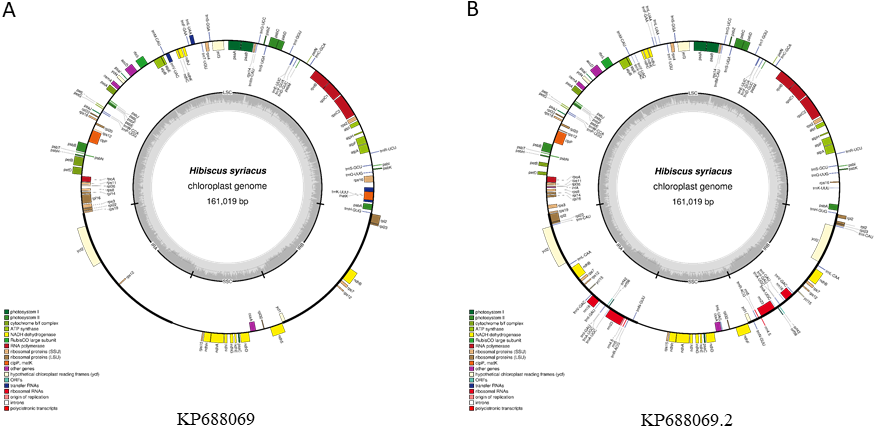


**Figure S5.** Chloroplast genome map and reannotation of chloroplast genome of *Hibiscus syriacus.*

The chloroplast maps were generated by Organellar Genome DRAW (<http://ogdraw.mpimp-golm.mpg.de/index.shtml>) ([Lohse et al., 2013](#_ENREF_3)) The gene inside and outside of the outer circle are transcribed in the clockwise and counterclockwise directions, respectively. Genes belonging to different functional groups are shown in different colors. The inner circle represents different regions of the chloroplast genome. IRA: invert repeat region A; IRB: Invert repeat region B; LSC: large single-copy region; SSC: small single-copy region. The line-chart in gray show GC content along the genome.
